# On the Immunity Enjoyed by the Stomach from Being Digested by Its Own Secretion during Life

**Published:** 1863-11

**Authors:** Frederick W. Pavy


### ON THE IMMUNITY ENJOYED BY THE STOMACH FROM BEING DIGESTED BY ITS OWN SECRETION DURING LIFE.

By FREDERICK W. PAVY, M. D.

The author referred to the communication by John Hunter "On the Digestion of the Stomach after Death," published in the "Philosophical Transactions" for 1772. In this communication Hunter notices that in occasional instances, especially in persons who have died of sudden and violent deaths, the stomach is found on inspection to have undergone solution, to the extent of perforation, from the action of its own secretion upon it. Hunter considered that this could only have taken place after death; and to account for why the same occurrence did not ensue during life, he adduced the living principle as constituting the protecting agent. The fact that parts of living animals, as shown by Claude Bernard of Paris, are susceptible of digestion when introduced through a fistulous opening into a digesting stomach, proved that Hunter's explanation does not stand the test of experiment. The author corroborated Bernard's results upon frogs, and referred to an experiment in which he had also obtained the digestion of the extremity of the ear of a living rabbit.

The view at present most generally entertained is, that the epithelial lining or mucus protects the stomach from undergoing digestion during life. This it is supposed is acted upon and dissolved, but being as constantly renewed, the stomach escapes injury. There being no longer the power of producing epithelium after death, accounts for the occurrence of the solution that may then be observed.

To test this view, the author removed a patch of mucous membrane about the size of a crown piece from the stomach of the dog. Food was afterwards digested without, however, the denuded stomach showing the slightest sign of attack. It thus appearing that the stomach resisted digestion notwithstanding the assumed protecting layer had been removed, it became evident that something besides the epithelial lining was required to account for the security enjoyed.

Seeing that the question was still open for explanation, the following was the view propounded by the author. The existence of acidity, it was first remarked, is an absolutely essential condition for the accomplishment of the act of digestion. During life the walls of the stomach are most freely permeated by a current of alkaline blood. Under such circumstances it would appear impossible that any digestive action could be effected. There would be one condition that would neutralize the other. Acidity is needful for digestion, and alkalinity is a constant character of the blood. As long, therefore, as so free a circulation of this alkaline fluid should be maintained (and this happens to be one of the necessary conditions of life), the stomach will be supplied with a source of protection competent to afford it the security from attack by its own secretion that it enjoys.

Digestion of the stomach may be effected after death, because the blood, being then stagnant, is incapable of offering the barrier produced by a circulating current.

Experiments were mentioned in which the circulation through the stomach had been arrested during life so as to imitate the condition, as far as the stomach was concerned, that exists after death. Although this was effected whilst the process of digestion was actively proceeding, yet it was only in some cases that the mucous membrane of the stomach was attacked. On repeating the experiment, however, having previously introduced a dilute non-corrosive acid (the phosporic and citric were the acids employed) into the stomach, the result was solution and perforation in a short space of time.

The author had expected, when he commenced his experiments, to have obtained the same result upon arresting the circulation through the stomach as occurs after death; but it became evident to him on reflection that although the circulation through the stomach may be stopped by ligatures during life, yet the conditions are not thereby rendered completely identical with those prevailing after death. There is still a circulation all around the stomach, and from the facility with which the permeation of fluids takes place, a certain amount of counteractive influence would still be exerted. By the artificial introduction, however, of an acid into the cavity of the stomach before its vessels were ligatured, the surrounding circulation became inadequate to afford the required neutralizing power, and perforation therefore quickly resulted.

It did not appear to the author that the digestion of the living tissues of animals referred to in the first part of his paper formed any valid objection to his view. In the case of the frog's legs, he considered it might be fairly taken that the amount of blood possessed by the animal would be inadequate to furnish the required means of resistance. In the case of the rabbit's ear, the vascularity of the part being so much less than that of the walls of the stomach, he thought there was nothing unreasonable in conceiving that, whilst the one might receive protection through the circulating alkaline current, the other might be unable to resist attack. There was no comparison between the position of the stomach and that of the rabbit's ear and the question, according to his view, resolved itself into degree of power possessed by the acidity of the contents of the stomach on the one hand, and the alkalinity of the circulating current on the other.

The author concluded by adducing experimental evidence to show that pepsine was contained in the walls of the stomachs of persons who had died from severe diseases, as well as in the normal fasting and digesting stomach.—*Proceedings of the Royal Society*, *Jan.*, 1863.

### CRUST OF BREAD.

M. Barral has presented to the Academy of Sciences some remarks of much interest concerning the crust of bread and the gluten contained in it. He had recently shown that, when equally dried, the crust of bread is more highly azotized than the crumb; and he also showed that the crust was more soluble than the crumb in water. M. Payen had, it is true, previously pointed out this greater solubility of the crust, and had ascribed it to the conversion of the starch into dextrine during the baking. But M. Barral's experiments show another important fact. "If," he says, "we exhaust with water an equal quantity of dry crumb and dry crust of bread, we find that the soluble portion of the crust consists of from 7 to 8 per cent. of nitrogen, whilst the soluble portion of the crumb contains only from 2 to 3 per cent. The greater solubility of the crust, consequently, depends upon the transformation which its gluten has undergone under the direct action of the 200° to 220° heat of the oven. The soluble portion of the crust is more highly azotized than the juice of meat." M. Barral added, that he was still engaged with his experiments, which he hoped would throw some new light on panification.—*British Med. Journal*, July 11, 1863.

